# Mental health of parents of children and adolescents who require special health care

**DOI:** 10.1590/0034-7167-2023-0457

**Published:** 2024-07-29

**Authors:** Eliana Roldão dos Santos Nonose, Rosane Meire Munhak da Silva, Eliane Tatsch Neves, Débora Falleiros de Mello, Adriana Zilly, Aline Cristiane Cavicchioli Okido, Regina Aparecida Garcia de Lima

**Affiliations:** I Universidade Estadual do Oeste do Paraná, Hospital Universitário do Oeste do Paraná. Cascavel, Paraná, Brazil; II Universidade Estadual do Oeste do Paraná. Foz do Iguaçu, Paraná, Brazil; III Universidade Federal de Santa Maria. Santa Maria, Rio Grande do Sul, Brazil; IV Universidade de São Paulo, Escola de Enfermagem de Ribeirão Preto. Ribeirão Preto, São Paulo, Brazil; V Universidade Federal de São Carlos. São Carlos, São Paulo, Brazil

**Keywords:** Mental Health, Country, Health Care, Pediatric Nursing, Health Vulnerability, Salud Mental, Padres, Atención Sanitaria, Enfermería Pediátrica, Vulnerabilidad en Salud

## Abstract

**Objective:**

To identify the manifestations presented by parents of children and adolescents who require special health attention that can impact their mental health.

**Methods:**

exploratory, qualitative research, based on the concept of vulnerability, with data collection carried out through interviews with 18 parents of children and adolescents with special health care needs, hospitalized in the pediatric ward of a hospital in Paraná, between May/2017 and May/ 2018. Data analyzed by inductive thematic analysis.

**Results:**

parents experienced situations of vulnerability when providing care at home, with repercussions on their mental health, expressed by manifestations of lack of protection, anxiety and depression.

**Final considerations:**

It is important that health professionals seek to expand actions to promote care and reduce situations that generate threats, insecurities, concerns and damage to the health of parents, which can impact and further weaken care for children and adolescents who need attention especially health.

## RESUMEN

**Objetivo:**

identificar las manifestaciones presentadas por los padres de niños y adolescentes que necesitan atención especial en salud que puedan afectar su salud mental.

**Métodos:**

Es una investigación exploratoria, cualitativa, basada en el concepto de vulnerabilidad, con datos examinados mediante el análisis temático inductivo y recolectados a través de entrevistas a 18 padres de niños y adolescentes con necesidades de cuidados especiales, internados en la unidad de pediatría de un hospital de Paraná, entre mayo de 2017 y mayo de 2018.

**Resultados:**

los padres experimentaron situaciones de vulnerabilidad en el ejercicio del cuidado domiciliario, con repercusiones en su salud mental, expresadas por manifestaciones de desprotección, ansiedad y depresión.

**Consideraciones finales:**

es importante que los profesionales sanitarios busquen ampliar sus acciones para promover el cuidado y reducir las situaciones que generen amenazas, inseguridades, preocupaciones y daños a la salud de los padres y perjudiquen el cuidado de los niños y adolescentes que necesitan atención especial.

## INTRODUCTION

Over time, technological and scientific advances in the field of health, especially in neonatal and pediatric wards, have contributed to increase the survival rates of premature children, those with low birth weight, congenital malformations and chronic diseases^(1)^. As a result of this process, there was an increase in demands for differentiated care and dedication from families to care for these children^(2)^.

In the United States of America (USA), this group has been studied since the 1980s and in 1998 they were called Children with Special Health Care Needs (CSHCN)^(3)^. In Brazil, the translation of the term for *Crianças com Necessidades Especiais de Saúde (CRIANES)* dates back to 1999^(4)^, maintaining the same definition, in free translation. Recently, the translation was updated for Children and Adolescents Who Need Special Health Attention^(5)^.

Children and adolescents who require special health care are characterized by the uniqueness and complexity of care and are classified according to their needs and not by medical diagnosis. These needs may be: continuous use of medication, use of medical, mental health or psycho-pedagogical services other than children in general, presence of functional limitation, need for rehabilitation therapy, treatment or counseling for emotional, developmental or behavioral problems^(1,6)^. This care represents challenges not only for health professionals, but above all for families and caregivers.

It is important to highlight that the main caregiver is in the family, since health professionals are temporary, but the family, theoretically, is a constant. In this context, it is family care that maintains the survival and quality of life of its members, justifying including the family as a unit of care and worthy of professional attention^(7)^.

Studies have shown that parental caregivers of children and adolescents who require special health care were twice as likely to seek health services, due to emotional exhaustion and symptoms of anxiety and depression^(8-10)^. Mental health refers to a state of well-being that allows people to deal with stressful daily experiences, far beyond the absence of mental disorders, as each individual experiences experiences differently, with varying degrees of difficulty and suffering^(11)^.

It is understood that caring for a child or adolescent who requires special health care has implications for the daily life and mental health of parental caregivers, placing them in a vulnerable condition^(12)^. This process can lead to psychological or physical maladjustment, with depressive symptoms in caregivers, especially if there is no active social support network contributing to care and helping to overcome vulnerabilities^(12-13)^.

Therefore, it is important to know the aspects that imply coping with a painful process in the face of an unplanned situation, generally marked by parents’ lack of preparation in care, concerns, doubts and resistance to accepting their child’s illness. In this study, the concept of vulnerability^(12)^ was important, considering that the context in which the family is inserted is fundamental for establishing adequate planning of health actions, aimed at promoting their physical and mental well-being.

## OBJECTIVE

To identify the manifestations presented by parents of children and adolescents who require special health care that can impact their mental health.

## METHODS

### Ethical aspects

This research was approved by the Research Ethics Committee of the Ribeirão Preto Nursing School of the University of São Paulo and complied with Resolution 466/2012 of the National Health Council. Participants were informed about the objectives of the study and procedures for collection of data and then, upon agreeing to participate, signed the Free and Informed Consent Form. There was no refusal from any participant.

To guarantee anonymity and enable the identification of the role of that family member, it was decided to carry out the coding using the role in the family nucleus, followed by the numerical identification assigned in order of participation (Ex.: Mother 1; Father 2), and so on. In excerpts from the interviews, the names of children and adolescents were replaced by the letter (C) as a child and by the letter (A) as a teenager, followed by the same number that represents the order of parental participation (Ex.: C1 Mother1, A10 Father10).

### Theoretical-methodological framework and study design

Exploratory study, with qualitative data analysis, based on the conceptual framework of vulnerability^(12)^. This concept, in the field of health, is related to the understanding of preventive practices that are articulated in the individual, social and programmatic components. Analyzed in an integrated way, these components can contribute to the recognition of care demands, with a view to comprehensiveness and the transformation of health practices.

The study was reported in accordance with the recommendations for developing qualitative research from the Consolidated Criteria for Reporting Qualitative Research (COREQ).

### Study Setting

The research was carried out in the Pediatric Inpatient Units of a University Hospital, called Pediatrics and Pediatric Intensive Care Unit. The institution is a reference for care for high-risk pregnancies and also for newborns, children and adolescents at risk in the west of the state of Paraná.

### Data Sources

The participants, selected intentionally, were 16 mothers and two fathers of children and adolescents hospitalized between May 2017 and May 2018 and who, initially, were classified according to the presence of any need for special health care, according to the version Brazilian version of the Children with Special Health Care Needs Screener© (CS©)^(6)^ instrument.

The inclusion criteria were being a regular companion of the child or adolescent, in order to share information in greater detail and being over 18 years old. As exclusion criteria, parents who, as recommended by the health team, demonstrated some cognitive deficit that would impair their participation and who had little knowledge about the child or adolescent’s daily life, or who did not speak Portuguese, preventing communication between participant and researcher.

### Data collection and organization

The technique chosen for data collection was the semi-structured interview, carried out in the hospital environment, in a private space, according to prior scheduling and participant availability, as the presence of another caregiver member was necessary to accompany the child or adolescent while the interview took place.

After collecting identification and sociodemographic data, the interview began, guided by the following question: Tell me about the impact of caring for your child on your daily life and that of your family. The interviews were recorded in audio, lasting an average of 40 minutes, and later transcribed in full by the researcher, who is a nurse, with professional experience in the area of children’s health. Even so, a pilot interview was carried out to adjust the script and train the researcher, included in the present study. A field diary was used to record observations considered relevant to the research and not capable of being recorded through recording, such as gestures, postures, facial expressions and crying. Participants were also offered to forward the transcription of the interviews to give their consent to the content.

The collection ended at the moment the participants’ information began to be repeated, without generating new information and the data allowed responding to the study objectives and enabled the understanding of the phenomenon of interest^(14)^.

### Data Analysis

The Inductive Thematic Analysis^(15)^ method was used to analyze the data, organized into six stages. Step 1 was the moment of immersion and familiarization with the data. After the interviews, full transcriptions were carried out and, with the reading and re-reading of the data obtained, initial ideas about the themes emerged. The elaboration of the initial codes was carried out manually in step 2, highlighting the main characteristics of the data in a systematic way, identifying the relevant data for each code. In step 3, the codes were grouped into potential themes, gathering relevant data for each potential theme, and in step 4, the identified themes and their validity in relation to the codes and data were reviewed. It was the time to refine the themes. The definition and naming of themes was carried out in stage 5, with a view to meeting the research objectives. Finally, in step 6, the final report was prepared, relating the data extracts that gave rise to the themes with the literature findings and the theoretical framework of vulnerability^(12)^. The entire process was carried out by six researchers, with the first and sixth authors identifying the themes and carrying out the analysis. The other authors certified and validated them.

## RESULTS

The study participants were 16 mothers and two fathers of children and adolescents in need of special health care, aged between 20 and 52 years and with education between two and 16 years. Regarding marital status, 14 declared themselves married, two single and two divorced. Regarding occupation, 14 reported taking care of the home, two were employed and two were self-employed; ten declared that they did not receive benefits from the government and the rest reported receiving the Continuous Payment Benefit. Three described having one child (only the child in need of special health care), seven with two children, four with three children, two with four children and two with five children. It is important to highlight that five participants did not receive help to care for their child.

Among the children and adolescents participating in the study, aged between eight months and 13 years and 7 months, nine were male and nine were female. Regarding the origin of special health needs, these were related to congenital perinatal causes (10), followed by acquired perinatal causes (5) and special needs due to causes acquired after one year of age (3). These needs caused demands for care beyond those presented by children of the same age, including technological (13), medication (18), developmental (18) and modified usual care (18) or the sum of all (13). Five children presented three types of care demands.

The families’ narratives showed that their child’s illness triggered vulnerable manifestations and experiences that impacted their mental health, expressed in the following themes: Attributes of the tiring routine; Guilt for the son’s illness and death hovering around his life.

### Attributes of a tiring routine

The routine of caring for a child or adolescent who requires special health care can be difficult and tiring, placing an overload on the family caregiver, who sometimes gives up their personal and social life for the care. These situations can increase stress and threaten mental health, according to reports:

*For my life, I’m pretty private. Now I have no more life, I live his life* [C3]. (Mother 3)

*Our routine have changed a lot because it was special, so we isolated ourselves a lot* [...]. *The routine is all about my daughter first and us second. While she doesn’t wake up, no one does anything, not breakfast, or anything, so as not to disturb her sleep. Only after she wakes up do we move on with our lives* [...]. *Our routine is like this, first her, then us.* (Mother 6)

*It’s a tiring routine, there’s no point saying that it doesn’t get tiring, it’s tiring, because it’s not just the body, it’s not just the mother’s physique, because there’s no point saying that the father takes care, because the father doesn’t take care.* (Mother 7)

Linked to overload, many participants mentioned a weakened family social network, little participation or even absent in the process of caring for children and adolescents. This fact signals isolation and lack of support to face this new experience:

*Almost no one goes to the house, just my mother, but her support is just to look, because she doesn’t want to do anything* [C1]*. My husband doesn’t move either, he’s afraid. Everyone is afraid that they will die by their hands* [...]. *It’s just the two of us and God.* (Mother 1)

*No one in the family was ready, no one ever wanted to learn how to vacuum. My mother-in-law sometimes stays with her* [C9], *when we go, for example, to the mall or the market. She follows her diet, she turns her sideways, she changes her diaper, but medications, suctioning, these things are me and the caregiver who stays from time to time.* (Mother 9)

Prejudice and discrimination resulting from stigma, associated with individual characteristics, materialized in behaviors of rejection and exclusion, resulting in separation from family and friends, as well as, sometimes, the breakdown of emotional relationships. Having a child who needs special health care is already painful and non-acceptance by society, or even the family, makes vulnerable parents suffer even more, expressing sadness and indignation:

*His family* [husband`s] *is even worse, they say that my daughter* [A6] *is defective.* (Mother 6)

*Family, no one cares, they don’t even care to know how we are. I think it’s prejudice they have towards special children* (Mother 8)

*Her father* [A11] *said she was a burden to him, that he couldn’t take care of her, that he wouldn’t admit having a daughter like that. God sent her to me like this, I will take care of her. Sometimes people ask me: how can you deal with this?* (Mother 11)

*My neighbors and friends, most of them, especially my friends from the good times, when we attended barbecues almost every week, moved away when times were difficult.* (Father 15)

Given the care demands presented by children, the reports also pointed to the need to abandon employment to dedicate themselves exclusively to care:

*Then I stopped working, I stayed with him* [A5]*, he went to APAE* [Association of Parents and Friends of the Exceptional]*, then it all started.* (Mother 5)

*When he* [A10] *was born, I worked as a cook, so we had to make a change, I stopped working to take care of him.* (Father 10)

*It changed our lives a lot, I stopped working to dedicate time to my son.* (Father 15)

*It changed completely. Before he was born, I worked, I did dressmaker. After he* [C16] *was born, impossible. With a tracheostomy, I can’t leave him in the room and sew it up and there isn’t even time. I have to take him to physiotherapy, just to give him a bath, it takes almost an hour, to change the little thing* [cannula] *of the tracheostomy, the equipment and those things.* (Mother 16)

Situations of parental illness such as symptoms of anxiety and depression, given the difficulties and clinical conditions presented by their children, were present in the families’ daily lives. During the process of living with the disease, emotions surfaced, resulting in conflicting or even contradictory attitudes towards the disease. Even those who adopted an active coping stance experienced moments of despair, sadness and depression:

*Most of my colleagues say: Oh how you’ve changed, you’re no longer that smiling girl, that happy girl, that girl who shined. But I don’t have any shine anymore. I think I’m already crying inside, to get it out, sometimes it’s difficult.* (Mother 3)

*There are times when I cry in secret, because it’s not easy. I feel more like this because of his suffering* [C16]*, because I know he suffers, poor thing, he gets all hurt with each hospitalization, that’s what hurts me, seeing him suffer. I hold him in my arms and cry, I wish it were different for him, that he was happier.* (Mother 16)

*I became depressed, anxious, I didn’t feel like doing anything and I gained a lot of weight.* (Mother 17)

*Before it was just joy, now in quotes it’s more sadness. I don’t see the fun in things anymore. In my service they charge a lot, because I worked always smiling, singing and now they say that every now and then they catch me in the corner crying, I don’t see the fun in anything anymore. I force a smile there and they say there’s no point in forcing it because they know I’m not cool, but I don’t see anything funny anymore.* (Mother 18)

From the participants’ reports, it is observed that the family members’ reaction had an influence on the emotional state of the caring parents:

*I feel alone with two children and my son* [C3] *unprotected, that’s how I feel, you know. Many of those who said: Oh my friend, everything will be fine before he was born, have abandoned me when I needed it. His father was the first, he was the one who left it all with me or at least pretended he did.* (Mother 3)

The physical and emotional overload and feelings of lack of protection experienced by parents of children and adolescents who require special health care, point to new challenges in taking care of themselves, their family and meeting the demands of their children, who live in an environment family member who may also have become ill and be submerged by vulnerable relationships, both individually and socially. It is noteworthy that a regular source of support, organized by the multidisciplinary team, could alleviate these vulnerable conditions.

### Blame for the child’s illness

The blame attributed to the child’s birth condition was evidenced as a feeling capable of leading to family conflicts, as there is an initial difficulty in dealing with the feelings and conflicts experienced:

*It’s very difficult to talk about it because, I’m a mother, I was a mother who, like, my other daughter was so normal* [...] *and my son* [C3] *isn’t now, I expected that, I expected it would be all easier. I feel guilty, you know. I did everything right, I went to all the* [prenatal] *appointments, I did all the tests, I took all the medicine, but something went wrong.* (Mother 3)

*His* [husband`] *family demands and says that it is my obligation to give him another child, because our daughter* [A6] *is defective. But he doesn’t want to, he’s afraid of having another child.* (Mother 6)

Maternal blame and the uncertainty of the reasons that led to the child’s condition influenced family daily life, suggesting manifestations of suffering and attitudes that undermined confidence and autonomy:

*Then he* [the child’s father] *said: the boy is there because of you, that my son got sick like this because of you. I said: it’s my fault, but tell me what I did wrong? What mistake did I make because he had this syndrome, because he had hydrocephalus?* (Mother 3)

*I found myself alone. When I told him* [husband] *that the doctor gave the diagnosis, that she* [A11] *had a clot in her brain, that she couldn’t operate, he said: that’s your fault.* (Mother 11)

*When I found out that she* [C12] *had this problem, I was very scared, I was in a state of shock, so I looked at her and just cried.* (Mother 12)

*He* [doctor] *told me that he had toxoplasmosis and that he was going to send me to Cascavel* [...] *and when he* [C13] *was born we didn’t know how serious it was, it was a blow, a shock.* (Mother 13)

On the other hand, although the participants expressed emotional problems, in their own way, they sought to appear strong and confident in caring for their child:

*He* [husband] *asks me if I’m not suffering, because I don’t cry in front of my husband, I act very tough. He says: Don’t you suffer? Doesn’t it hurt at all? I say no, because I learned to be like this, it has to be like this, you can’t cry around her, but deep down, inside she’s already crying. I wait for him to sleep to cry, I go to the bathroom when he’s sleeping, at work, but in front of him and her my motto is don’t cry.* (Mother 18)

Guilt for the child’s condition is a feeling that impacts the lives of families, weakening relationships and the care of the child itself. Therefore, family support and involvement in care is important to seek emotional balance and avoid the intensification of existing individual vulnerability.

### Death hovering around life

The perspective that fragile children are at risk of dying prematurely was present in the participants’ reports, given the clinical instability, the uncertainty of the diagnosis, the long periods of hospitalization and the need for readmissions:

*So, he* [doctor] *said he was going to die at any moment. When they found out about the diagnosis they confirmed, it was even worse. And I was scared, because he* [doctor] *said: You’re going to have a condition and you’re going to die.* (Mother 1)

*Our! It’s difficult when you have to leave your child. One day already hurts, one month, my God, it’s an eternity, two more months pass and it’s crazy, three months you’re out of your mind. You are out of your mind. He is such a strong child, strong, very strong, but he is a child that I look at who is tired, you know, that I see that* [...] *I don’t even know how to explain it* [...] *he* [C3] *has been there for several several times on the brink of death.* (Mother 3)

*When he* [C7] *gets sick and we don’t know what’s happening, it’s still scary, it’s not 100% safe, at the beginning it was very complicated, it was scary, insecure.* (Mother 7)

*She* [doctor] *said that my daughter was going to die at one year and three months, I was desperate because she was almost nine months old. At one year and three months old, she was admitted to the hospital very seriously, everyone was in a state of shock, thinking she was going to die. She spent one month and 25 days in the ICU* [Intensive Care Unit], *seven months in hospital.* (Mother 12)

The fear of losing their child was constant and affected the parents’ daily lives:

*Now with the fan I can get some sleep, because then it lets me know when she* [A4] *is not well. Before, I found it more difficult, because I was afraid of going to sleep and waking up and she* [A4] *wouldn’t wake up anymore.* (Mother 4)

The reports expressed fears related to individual and biological vulnerabilities and these feelings increased concerns and feelings of apprehension about the health of other family members, generating more suffering in the parents:

*I also think about my other son, because if it is genetic, me, my husband, someone else have it.* (Mother 1)

*When I was 6 months pregnant, my other son died at one year and eight months old, he also had a syndrome that was not diagnosed, that’s why I’m chasing it, I’m asking everyone to see if there’s anything, because the problem I think it could be the same problem that the other one had.* (Mother 2)

## DISCUSSION

The present study shows that the health condition of a child or adolescent that requires special attention awakens vulnerable feelings and experiences for parents, who walk a path of fear and loneliness, which can impact their mental health.

The vulnerabilities of families of patients who require complex and differentiated care for a long period have hospital discharge as a milestone, considering the various changes implemented in family daily life. Studies identify that, at the time of discharge, there is a transfer of responsibility for care to the family who do not have the necessary knowledge to exercise this care in the home environment^(2,7,16)^.

Caring for a child in this dimension requires a reorganization of the domestic routine, and this changes the family dynamics, as there will be a need for a full-time family caregiver to meet the countless demands arising from the chronic condition, configuring itself as an exhausting and overload generator. The accumulation of these care demands over time can lead many parents to experience poor mental health outcomes, with higher levels of stress, anxiety and depression^(8-9,17)^.

It is important to highlight that this overload is not only related to the demands of care, but to feelings of concern about the child’s illness, impotence and lack of knowledge about how to care for the child^(7,17-18)^. Although the chronic condition affects the entire family, in general, the mother, as she is the one most involved in care, starts to live for her child, giving up her own life^(19)^. The maternal figure, as the main caregiver of children with special health care needs, was evidenced in numerous studies^(18-23)^, as well as the need for the main family caregiver to abandon employment^(22-23)^, intensifying social vulnerability, both due to financial disadvantage and the lack of socialization with other individuals.

In this context, it is understood that, faced with the child’s diagnosis, the family is faced with vulnerable relationships and presents signs of suffering in many dimensions, be it physical, psychological and social, which can have an impact on the child’s care^(2)^. This condition of vulnerability and the need for emotional balance impact the mental health of parents, who sometimes seek psychosocial and medication support^(24)^.

To meet their child’s health care demands, some parents needed to abandon their social life, in an attempt to provide the necessary care, but also to protect their child from social embarrassment and humiliation, due to the stigma and prejudice still very present in society^(25)^. Prejudice was highlighted by the family members themselves in this investigation, and this attitude led them to withdraw and face the situation alone. These situations can make them more vulnerable to developing psychological distress and mental disorders^(26)^.

A study confirmed these findings by finding that 82.7% of mothers had depression and of these, 26.6% had severe depression. It was observed that the greater the child’s functional limitation and the younger the age, the greater the level of depression among caregivers and that depression had a negative impact on the caregivers’ quality of life^(23)^. Furthermore, recent research has identified that family caregivers can increase the level of stress, anxiety and depressive symptoms^(10,27)^, and these problems may require psychopharmacological treatment and psychological support^(24)^. Furthermore, this situation can be considered as a sign of probable exposure of the child to adverse situations, of insufficiencies in care actions, due to the limitations of caregivers due to their illness^(23,28)^. Other studies highlighted that family members can even be affected physically (sleep deprivation, tiredness), mentally and financially, with a negative impact on the quality of ongoing care and the functioning of the family as a whole^(13,17,25)^.

From this perspective, it is important that health professionals have a different look at these family members who have also become ill and need care^(7,25)^, with priority given to welcoming, qualified listening, support and promotion and protection actions. health^(17,29)^. It is necessary to avoid creating new vulnerabilities, especially for the main caregiver, represented by the mother in this study. Actions and practices that are not sensitive to the singularities of these mothers and their intersubjective contexts can reproduce stigmas and gender discrimination^(12)^.

As observed in the results, there is a constant need for support for mothers who care for a child or adolescent who needs special health care, support not only from their spouse, but also from other family members, friends and health professionals.

The feeling of guilt for the child’s illness, observed in this research, was negatively associated with happiness and served as a mediator between attachment anxiety, support and happiness. This feeling was also found in a study with mothers of children with Congenital Syndrome caused by the Zika virus^(30)^ and cerebral palsy^(23)^. The family, mostly represented by the mother, in search of answers, attributes responsibility for the child’s clinical condition. A feeling of guilt and thoughts of self-blame can lead to low self-esteem^(23)^.

A study also found that the arrival of a child with a disability frustrates expectations and generates feelings of failure, guilt and sadness related to mourning the loss of the idealized perfect child^(25)^. Having suffered losses, such as the death of another family member, no matter how much time has passed, makes the family remember and feel frightened by the possibility of it happening again. Furthermore, anxiety and the fear of losing the child emerge as feelings that distress parents of children with multiple disabilities and are caused by the perception of vulnerability and the need to protect their children^(25)^.

The demands generated by this context increase parents’ anxiety, depressive symptoms, stress and tension reactions, feelings of impotence and helplessness, which can culminate in social isolation and worsen the family situation^(10,24)^. Therefore, it is important that health professionals are prepared to guide and support them in carrying out daily care, so that anxiety and fear are minimized, promoting safety for quality care for themselves and their child and which, in addition, can contribute to reducing readmissions^(31)^.

The use of specific adaptive coping strategies (i.e., emotional support, positive reframing, and acceptance) may be promising to support parents in vulnerable situations. Support interventions through psychoeducation and implementation of coping behaviors can minimize the impact that social isolation and negative cognitive evaluations may have on the mental health outcomes of parents and children, expanding the ways to care for these families^(10)^.

The way people react to the diagnosis of a chronic disease is determined by their living conditions, meanings attributed to the disease, access to resources available to control the health-disease process, as well as the social network and support received. Therefore, the recognition and identification of vulnerability, by the interprofessional team, present in the lives of children and adolescents who require special health care, as well as their families, is important, as it can contribute to the recognition of health demands. care for both with a view to comprehensive health care^(7,32)^.

Considering the harmful effects that parents’ mental health can have on the quality of care provided to their child, timely and effective interventions are necessary, with a view to balancing the responsibilities and demands that accompany the care of a child or adolescent with care needs. special to health.

### Study limitations

The fact that the study was conducted in the hospital environment may have limited knowledge about the needs for home care. Although families have reported it, it is necessary to consider that when entering the home environment, new vulnerabilities to care may arise, implying the need for professional and community support to provide care with safety and autonomy. In this sense, expansion to other investigations is needed, based on observation of care and interventions to reduce the vulnerabilities of these families in home care.

### Contributions to the area of nursing, health and public policies

The study contributes to the design of strategies to improve the care of families of children and adolescents who require special needs, by considering and identifying signs that can intensify or create vulnerabilities, especially in the emotional/individual and social dimension, a condition that can weaken the care of children and adolescents at home, who need care, affection and dedication from their parents to grow and develop in a healthy way. To achieve this, a transformation is needed in health work processes, with actions sensitive to the singularities of families, especially mothers, who are in great need of care as they are vulnerable in this entire context.

## FINAL CONSIDERATIONS

The present investigation identified that parents of children and adolescents who require special needs experience moments of individual and social vulnerability when providing care at home, due to overload, isolation and sadness, mainly because they do not receive support from their family members. Sometimes, these same family members blame them for their child’s illness, leading them to believe that they are in fact to blame for their child needing special health care. Still, the fear of their son’s death haunts their lives. Such experiences can contribute to the occurrence of manifestations that compromise mental health, expressed by feelings of lack of protection, anxiety and depression.

Considering the responsibility of parents and the constant need for attention from their children, it is important that health professionals seek to expand promotional actions with the family as the unit of care, with a view to recognizing and reducing situations that generate new vulnerabilities, or that is, threats, uncertainties, concerns and damages, which can impact and weaken the care for children and adolescents with special health care needs. In summary, it is essential to offer multidisciplinary support to caring parents, especially in the emotional and social dimension, to promote their mental health.
