# Supplementary material for: Mental health of parents of children and adolescents who require special health care
Source: Rev Bras Enferm. 2024 Jul 29;77(3):e20230457. doi: 10.1590/0034-7167-2023-0457 (PMC11290732; doi:10.1590/0034-7167-2023-0457)
Supplement: Translation

# Translation: Saúde mental de pais de crianças e adolescentes que necessitam de atenção especial à saúde

**DOI:** 10.1590/0034-7167-2023-0457pt

**Authors:** Eliana Roldão dos Santos Nonose, Rosane Meire Munhak da Silva, Eliane Tatsch Neves, Débora Falleiros de Mello, Adriana Zilly, Aline Cristiane Cavicchioli Okido, Regina Aparecida Garcia de Lima

**Affiliations:** I Universidade Estadual do Oeste do Paraná, Hospital Universitário do Oeste do Paraná. Cascavel, Paraná, Brasil; II Universidade Estadual do Oeste do Paraná. Foz do Iguaçu, Paraná, Brasil; III Universidade Federal de Santa Maria. Santa Maria, Rio Grande do Sul, Brasil; IV Universidade de São Paulo, Escola de Enfermagem de Ribeirão Preto. Ribeirão Preto, São Paulo, Brasil; V Universidade Federal de São Carlos. São Carlos, São Paulo, Brasil

**Keywords:** Saúde Mental, Pais, Atenção à, Saúde, Enfermagem Pediátrica, Vulnerabilidade em Saúde

## Abstract

**Objetivo:**

identificar as manifestações apresentadas pelos pais de crianças e adolescentes que necessitam de atenção especial à saúde e que podem impactar a sua saúde mental.

**Métodos:**

pesquisa exploratória, qualitativa, fundamentada no conceito de vulnerabilidade, com coleta de dados realizada por meio de entrevistas com 18 pais de crianças e adolescentes com necessidades de atenção especial, hospitalizadas em unidade pediátrica de um hospital do Paraná, entre maio/2017 e maio/2018. Dados analisados pela análise temática indutiva.

**Resultados:**

os pais experienciaram situações de vulnerabilidades para o exercício do cuidado no domicílio, com repercussões em sua saúde mental, expressas por manifestações de desproteção, ansiedade e depressão.

**Considerações finais:**

é relevante que profissionais de saúde busquem ampliar as ações para promover o cuidado e reduzir as situações que geram ameaças, inseguranças, preocupações e danos para a saúde dos pais, que podem impactar e fragilizar ainda mais o cuidado às crianças e adolescentes que necessitam de atenção especial à saúde.

## INTRODUÇÃO

Os avanços tecnológicos e científicos no campo da saúde, ao longo do tempo, especialmente, na área neonatal e pediátrica, possibilitaram o aumento das taxas de sobrevivência de crianças prematuras, com baixo peso ao nascer, com malformações congênitas e doenças crônicas^(1)^. Em decorrência desse processo, houve o aumento de demandas de cuidados diferenciados e da dedicação das famílias para o cuidado das crianças que apresentam tais condições^(2)^.

Nos Estados Unidos da América (EUA), esse grupo é estudado desde a década de 1980 e foi denominado, em 1998, como *Children with Special Health Care Needs* (CSHCN)^(3)^. No Brasil, a tradução do termo para Crianças com Necessidades Especiais de Saúde (CRIANES) é de 1999^(4)^, mantendo a mesma definição, em tradução livre. Recentemente, a tradução foi atualizada para Crianças e Adolescentes que Necessitam de Atenção Especial à Saúde^(5)^.

As crianças e os adolescentes que necessitam de atenção especial à saúde caracterizam-se pela singularidade e complexidade de cuidados e são classificados segundo suas necessidades e não pelo diagnóstico médico. Essas necessidades podem ser: uso contínuo de medicamentos, utilização de serviços médicos, de saúde mental ou psicopedagógicos para além das crianças em geral, presença de limitação funcional, necessidade de terapia de reabilitação, tratamento ou aconselhamento para problemas emocionais, de desenvolvimento ou comportamento^(1,6)^. Estes cuidados representam desafios não só para os profissionais de saúde, mas sobretudo para as famílias e cuidadores.

É importante destacar que o principal cuidador se encontra na família, visto que os profissionais de saúde são temporários, mas a família, teoricamente, é uma constante. Nesse contexto, o cuidado família mantém a sobrevivência e a qualidade de vida de seus membros, o que justifica a sua inclusão como unidade de cuidado merecedora de atenção profissional^(7)^.

Estudos mostraram que os cuidadores parentais de crianças e adolescentes que necessitam de atenção especial à saúde apresentaram duas vezes mais probabilidade de procurar por serviços de saúde, pelo desgaste emocional, com sintomas de ansiedade e depressão^(8-10)^. A saúde mental se refere a um estado de bem-estar que permite às pessoas lidarem com experiências cotidianas estressantes, muito além de ausência de transtornos mentais, visto que cada indivíduo vivencia experiências de forma diferente, com graus variados de dificuldade e sofrimento^(11)^.

Compreende-se que o cuidar de uma criança ou adolescente que necessita de atenção especial à saúde tem implicações na vida cotidiana e na saúde mental dos cuidadores parentais, inserindo-os em uma condição de vulnerabilidade^(12)^. Tal processo pode levar a desajustes de ordem psicológica ou física, com sintomas depressivos nos cuidadores, principalmente, se não houver uma rede de apoio social ativa contribuindo para o cuidado e ajudando a superar as vulnerabilidades^(12-13)^.

Desse modo, é relevante conhecer os aspectos que implicam no enfrentamento de um processo doloroso diante da situação não planejada, marcada, geralmente, pela falta de preparo dos pais nos cuidados, preocupações, dúvidas e a resistência em aceitar a condição de doença do filho. Neste estudo, o conceito de vulnerabilidade^(12)^ foi importante, por considerar que o contexto que a família está inserida é fundamental para que se estabeleça um planejamento adequado das ações em saúde, voltadas à promoção de seu bem-estar físico e mental.

## OBJETIVO

Identificar as manifestações apresentadas pelos pais de crianças e adolescentes que necessitam de atenção especial à saúde que podem impactar a sua saúde mental.

## MÉTODOS

### Aspectos éticos

A presente pesquisa foi aprovada pelo Comitê de Ética em Pesquisa da Escola de Enfermagem de Ribeirão Preto da Universidade de São Paulo e atendeu à Resolução 466/2012 do Conselho Nacional de Saúde. Os participantes foram esclarecidos sobre os objetivos do estudo e procedimentos para a coleta de dados e, em seguida, ao concordarem em participar, assinaram o Termo de Consentimento Livre e Esclarecido. Não houve recusa de nenhum participante.

Para garantir o anonimato e possibilitar a identificação do papel daquele membro da família, optou-se por fazer a codificação utilizando a função no núcleo familiar, seguida da identificação numérica atribuída por ordem de participação (Ex.: Mãe 1; Pai 2), e assim sucessivamente. Nos trechos das entrevistas, os nomes das crianças e adolescentes foram substituídos pela letra (C), quando criança e pela letra (A) quando adolescente, seguida do mesmo número que representa a ordem de participação dos pais (Ex.: C1 - Mãe1, A10 - Pai10).

### Referencial teórico-metodológico e tipo de estudo

O estudo é exploratório, com análise qualitativa dos dados, fundamentado no quadro conceitual de vulnerabilidade^(12)^. Este conceito se relaciona, no campo da saúde, com a compreensão das práticas preventivas que se articulam nos componentes individual, social e programático. Analisados de forma integrada, os componentes podem contribuir no reconhecimento das demandas de cuidado, com vistas à integralidade e à transformação das práticas em saúde.

O estudo foi reportado de acordo com as recomendações para elaboração de pesquisas qualitativas do *Consolidated Criteria for Reporting Qualitative Research* (COREQ).

### Cenário do estudo

A pesquisa foi desenvolvida nas Unidades de Internação Pediátrica de um Hospital Universitário, nominadas Pediatria e Unidade de Terapia Intensiva Pediátrica. A instituição é referência para o atendimento à gestação de alto risco e, também, no atendimento de recém-nascidos, crianças e adolescentes de risco do oeste do estado do Paraná.

### Fonte de dados

Os participantes, selecionados intencionalmente, foram 16 mães e dois pais de crianças e adolescentes hospitalizados entre maio de 2017 a maio de 2018 e que, em um primeiro momento, foram classificados segundo a presença de alguma necessidade de atenção especial à saúde, segundo a versão brasileira do instrumento *Children with Special Health Care Needs Screener^©^* (CS^©^)^(6)^.

Os critérios de inclusão foram: ser acompanhante habitual da criança ou do adolescente, de forma a compartilhar informações com maior teor de detalhes, além de ser maior de 18 anos. Como critérios de exclusão, adotou-se que seriam excluídos os pais que, por indicação da equipe de saúde, demonstrassem algum déficit cognitivo que prejudicasse a sua participação e que tivessem pouco conhecimento acerca do cotidiano da criança ou do adolescente, ou que não falassem o idioma português, impedindo a comunicação entre participante e pesquisadora.

### Coleta e organização dos dados

A técnica eleita para a coleta de dados foi a entrevista semiestruturada, realizada no ambiente hospitalar, em um espaço privativo, conforme agendamento prévio e disponibilidade do participante, pois era necessário a presença de outro membro cuidador para acompanhar a criança ou o adolescente enquanto a entrevista ocorria.

Após a coleta dos dados de identificação e sociodemográficos, teve início a entrevista norteada pela seguinte questão: *Me conte sobre o impacto do cuidado ao seu filho no seu cotidiano e de sua família.* As entrevistas foram gravadas em áudio, com duração média de 40 minutos, posteriormente, transcritas na íntegra pela pesquisadora, a qual é enfermeira, com experiência profissional na área da saúde da criança. Mesmo assim, foi realizada uma entrevista piloto para ajustes no roteiro e treinamento da pesquisadora, incluída no presente estudo. Utilizou-se um diário de campo para o registro de observações consideradas relevantes para a pesquisa e não passíveis de registro por meio da gravação, tais como, gestos, posturas, expressões faciais e choro. Foi também oferecido aos participantes o encaminhamento da transcrição das entrevistas para darem anuência quanto ao conteúdo.

A coleta foi encerrada no momento em que as informações dos participantes começaram a se repetir, sem suscitar novas informações e os dados permitiram responder aos objetivos do estudo e possibilitaram a compreensão do fenômeno de interesse^(14)^.

### Análise dos dados

Para análise dos dados, foi utilizado o método de Análise Temática Indutiva^(15)^, organizado em seis etapas. A etapa 1 foi o momento de imersão e familiarização com os dados. Após as entrevistas, realizou-se as transcrições na íntegra e, com a leitura e releitura dos dados obtidos, emergiram as ideias iniciais acerca dos temas. A elaboração dos códigos iniciais foi realizada, manualmente, na etapa 2, destacando-se as características principais dos dados de uma forma sistemática, identificando os dados relevantes para cada código. Na etapa 3, agrupou-se os códigos em temas potenciais, reunindo os dados relevantes para cada tema potencial e, na etapa 4, foram revisados os temas identificados e a validade deles em relação aos códigos e aos dados. Foi o momento de refinamento dos temas. A definição e nomeação dos temas foi concretizada na etapa 5, tendo em vista atender aos objetivos da pesquisa. Por fim, na etapa 6, elaborou-se o relatório final, relacionando os extratos de dados que deram origem aos temas com os achados da literatura e ao referencial teórico da vulnerabilidade ^(12)^. Todo o processo foi realizado por seis pesquisadoras, sendo que a primeira e a sexta identificaram os temas e procederam à análise. As demais autoras os certificaram e os validaram.

## RESULTADOS

Os participantes do estudo foram 16 mães e 2 pais de crianças e adolescentes com necessidade de atenção especial à saúde, com idades entre 20 e 52 anos e escolaridade entre dois e 16 anos. Sobre o estado civil, 14 se declararam casados, dois solteiros e dois divorciados. Sobre a ocupação, 14 informaram cuidar do lar, dois com vínculo empregatício e dois trabalhadores autônomos; dez declararam não receber benefício do governo e os demais informaram receber o Benefício de Prestação Continuada. Três descreveram ter um filho (apenas a criança com necessidade de atenção especial à saúde); sete com dois filhos; quatro com três filhos; dois com quatro filhos e dois com cinco filhos. Importante destacar que cinco participantes não recebiam ajuda para o cuidado do(a) filho(a).

Entre as crianças e adolescentes participantes do estudo, elas possuíam idades entre 8 meses e 13 anos e 7 meses, nove eram do sexo masculino e nove feminino. Com relação à origem das necessidades especiais de saúde, elas relacionaram-se às causas perinatais congênitas (10), seguida de causas perinatais adquiridas (5) e necessidades especiais por causas adquiridas após um ano de idade (3). Essas necessidades causaram demandas de cuidado para além daquelas apresentadas por crianças da mesma idade, incluindo os cuidados tecnológicos (13), medicamentosos (18), de desenvolvimento (18) e habituais modificados (18) ou a soma de todos (13). Cinco crianças apresentaram três tipos de demandas de cuidados.

As narrativas das famílias mostraram que a doença do filho desencadeou manifestações e experiências vulneráveis que impactaram sua saúde mental, expressas nos seguintes temas: “Atributos da rotina cansativa”; “Culpa pela condição de doença do filho” e “A morte rondando a vida”.

### Atributos da rotina cansativa

A rotina de cuidados com a criança ou adolescente que necessita de atenção especial à saúde pode ser difícil e cansativa, levando a sobrecarga do familiar cuidador, que por vezes, abdica de sua vida pessoal e social, para o cuidado. Tais situações podem potencializar o estresse e ameaçar a saúde mental, segundo os relatos:

*Para minha vida eu sou bem privada. Agora não tenho mais vida, eu vivo a vida dele* [C3]. (Mãe 3)

*Mudou bastante nossa rotina por ela ser especial, então, nós nos isolamos bastante* [...]*. A rotina é tudo primeiro a minha filha e depois nós. Enquanto ela não acorda ninguém faz nada, nem café da manhã, nem nada, para não atrapalhar o soninho dela. Só depois que ela acorda que tocamos a vida para frente* [...]. *Nossa rotina é assim, primeiro ela, depois nós.* (Mãe 6)

*É uma rotina cansativa, não adianta falar que não cansa, cansa, porque não é só o corpo, não é só o físico da mãe, porque não adianta falar que pai cuida, porque pai não cuida.* (Mãe 7)

Atrelada à sobrecarga, muitos participantes mencionaram uma rede social familiar fragilizada, pouco participativa ou até mesmo ausente no processo do cuidado da criança e do adolescente. O fato sinaliza o isolamento e falta de apoio para o enfrentamento dessa nova experiência:

*Lá em casa quase ninguém vai, só a minha mãe, mas, o apoio dela é só olhar, porque ela não quer mexer* [com a C1]*. Meu esposo não mexe também, tem medo. Todo mundo tem medo que morra na mão deles* [...]. *Somos só nós dois e Deus.* (Mãe 1)

*Da família ninguém se prontificou, ninguém nunca quis aprender a aspirar. A minha sogra às vezes que fica com ela* [C9], *quando vamos, por exemplo, ao shopping ou no mercado. Ela põe a dieta, ela vira de ladinho, ela troca a fralda, mas medicações, aspiração, essas coisas sou eu e a cuidadora que fica de vez em quando.* (Mãe 9)

O preconceito e a discriminação decorrentes do estigma, associados às características individuais, concretizaram-se em comportamentos de rejeição e exclusão, resultando em afastamento de familiares e amigos, bem como, algumas vezes, o rompimento de relações afetivas. Ter um filho que necessita de atenção especial à saúde já é algo sofrido e a não aceitação por parte da sociedade, ou até mesmo da família, faz com que os pais, vulnerabilizados, sofram ainda mais, manifestando tristeza e indignação:

*A família dele* [esposo] *é pior ainda, diz que a minha filha* [A6] *é defeituosa.* (Mãe 6)

*Família, ninguém dá bola, nem ligam para saber como que está. Acho que é preconceito que eles têm com criança especial.* (Mãe 8)

*O pai dela* [A11] *disse que ela era uma carga para ele, que ele não podia cuidar, que não admitia ter uma filha assim. Deus me mandou ela assim, eu vou cuidar dela. Às vezes me perguntam: como que tu consegues lidar com isso? Uma cruz para carregar!* (Mãe 11)

*Meus vizinhos e amigos, a maioria, principalmente meus amigos da época boa, de quando participávamos de churrasco quase toda semana, se afastaram na hora da dificuldade.* (Pai 15)

Tendo em vista as demandas de cuidado apresentadas pelos filhos, os relatos também apontaram para a necessidade do abandono do emprego para se dedicar exclusivamente ao cuidado:

*Daí eu parei de trabalhar, fiquei junto com ele* [A5], *ele ia na APAE* [Associação dos Pais e Amigos do Excepcionais]*, daí começou tudo.* (Mãe 5)

*Quando ele* [A10] *nasceu, eu trabalhava de cozinheiro, aí tivemos que fazer uma troca, eu deixar de trabalhar para cuidar dele.* (Pai 10)

*Mudou bastante nossa vida, deixei de trabalhar para dedicar ao meu filho.* (Pai 15)

*Mudou completamente. Antes dele nascer eu trabalhava, fazia corte e costura. Depois que ele* [C16] *nasceu, impossível. Com traqueostomia, não tenho como largar ele lá no quarto e costurar e nem dá tempo. Tenho que levar na fisioterapia, só para dar banho vai quase uma hora, trocar a coisinha* [cânula] *da traqueostomia, o equipo e essas coisas.* (Mãe 16)

Situações de adoecimento dos pais, como sintomas de ansiedade e depressão, diante das dificuldades e condições clínicas apresentadas pelos seus filhos, estiveram presentes no cotidiano das famílias. No decorrer do processo de viver a doença, emoções afloraram, resultando em atitudes conflitantes ou mesmo contraditórias, frente à doença. Até mesmo aqueles que adotaram uma postura de enfrentamento ativo experimentaram momentos de desespero, tristeza e depressão:

*A maioria das minhas colegas fala: Ai como você mudou, você não é mais aquela menina sorridente, aquela menina alegre, aquela menina que brilhava. Mas eu não tenho mais brilho. Acho que eu já choro por dentro, para botar para fora, às vezes é difícil.* (Mãe 3)

*Tem horas que eu choro escondida, porque não é fácil. Eu fico mais assim pelo sofrimento dele* [C16], *porque sei que ele sofre, coitadinho, ele fica todo machucado a cada internamento, isso que me dói, de ver ele sofrer. Pego ele no colo e choro, queria que fosse diferente para ele, que ele fosse mais feliz.* (Mãe 16)

*Fiquei depressiva, ansiosa, não tinha vontade de fazer nada e engordei muito.* (Mãe 17)

*Antes era só alegria, agora entre aspas é mais tristeza. Não vejo mais graça nas coisas. Lá no meu serviço eles cobram muito, porque eu trabalhava sempre sorrindo, cantando e agora eles falam que volta e meia eles me pegam pelos cantos chorando, não vejo mais graça em nada, em nada. Eu forço um sorriso lá e eles dizem que não adianta forçar porque sabem que não estou legal, mas não vejo mais graça em nada.* (Mãe 18)

Pelos relatos dos participantes observa-se que a reação dos familiares teve influência no estado emocional dos pais cuidadores:

*Eu me sinto sozinha com duas crianças e o meu filho* [C3] *desprotegida, é assim que eu me sinto sabe. Muitos dos que falaram: Ai amiga, vai dar tudo certo, antes dele nascer, me abandonaram quando eu precisei. O pai dele foi o primeiro, que foi o que viveu tudo comigo ou pelo menos fingiu que viveu.* (Mãe 3)

A sobrecarga física, emocional e os sentimentos de desproteção vivenciados pelos pais de crianças e adolescentes que necessitam de atenção especial de saúde apontaram para novos desafios para o cuidado de si, da família e para atender às demandas dos filhos, que vivem em um ambiente familiar que também pode ter adoecido e estar submerso por relações vulnerabilizadas, no componente individual e social. Ressalta-se que uma fonte regular de apoio, organizada pela equipe multiprofissional poderia tornar mais leve essas condições vulneráveis.

### Culpa pela condição de doença do filho

A culpa atribuída pela condição de nascimento do filho foi evidenciada como um sentimento capaz de levar a conflitos familiares, já que existe uma dificuldade inicial em lidar com os sentimentos e conflitos vivenciados:

*É bem difícil falar porque, eu sou uma mãe, eu era uma mãe que tipo, a minha outra filha foi tão normal* [...] *e o meu filho* [C3] *agora não é, eu esperei isso, esperei que ia ser tudo mais fácil. Eu me sinto culpada sabe. Eu fiz tudo certo, eu fui em todas consultas* [pré-natal]*, eu fiz todos exames, tomei todos remédios, mas alguma coisa deu errada.* (Mãe 3)

*A família dele* [esposo] *cobra e diz que é minha obrigação dar outro filho para ele, por nossa filha* [A6] *ser defeituosa. Mas ele não quer, ele tem medo de ter outro filho.* (Mãe 6)

A culpabilização materna e a incerteza dos motivos que levaram à condição da criança influenciaram o cotidiano familiar, sugerindo manifestações de sofrimentos e atitudes que abalaram a confiança e autonomia:

*Aí ele* [pai da criança], *fala assim: o menino está aí por sua culpa, que meu filho veio doente assim por sua culpa. Eu falo assim: é minha culpa, mas, me diz o que eu fiz de errado? Que erro eu cometi por ele ter essa síndrome, por ele ter hidrocefalia?* (Mãe 3)

*Me vi sozinha. Quando falei para ele* [esposo] *que o médico deu o diagnóstico, que ela* [A11] *tinha um coágulo no cérebro, que não tinha como operar, ele disse: isso é culpa tua.* (Mãe 11)

*Quando eu descobri que ela* [C12] *tinha esse problema, eu tive muito medo, eu fiquei em estado de choque, daí eu olhava para ela e só chorava.* (Mãe 12)

*Ele* [médico] *me falou que estava com toxoplasmose e que ia me encaminhar para Cascavel* [...] *e quando ele* [C13] *nasceu não sabíamos da gravidade, foi um baque, um choque.* (Mãe 13)

Por outro lado, embora os participantes tenham manifestado problemas emocionais, ainda a seu modo buscaram se mostrar fortes e seguros para o exercício do cuidado do filho:

*Ele* [esposo] *pergunta para mim se eu não sofro, porque eu não choro na frente do meu marido, eu me passo por muito durona. Ele fala: Você não sofre? Não te dói nada? Eu falo que não, porque eu aprendi a ser assim, tem que ser assim, você não pode chorar perto dela, mas no fundo, lá dentro já está chorando. Eu espero ele dormir para chorar, eu vou para o banheiro quando ele está dormindo, no serviço, mas na frente dele e dela meu lema é esse não chorar.* (Mãe 18)

A culpa pela condição do filho é um sentimento que impacta na vida das famílias, fragiliza as relações e o próprio cuidado do filho. Por isso, é importante o apoio e envolvimento da família nos cuidados para buscar o equilíbrio emocional e evitar a intensificação da vulnerabilidade individual já existente.

### A morte rondando a vida

A perspectiva de que os filhos frágeis estão sob riscos de morrer precocemente esteve presente nos relatos dos participantes, dada a instabilidade clínica, a incerteza do diagnóstico, os longos períodos de hospitalização e a necessidade de reinternações:

*Então, ele* [médico] *falava que ia morrer a qualquer momento. Quando souberam do diagnóstico que eles confirmaram, foi pior ainda. E eu com medo, porque ele* [médico] *dizia: Vai ter um quadro e vai morrer.* (Mãe 1)

*Nossa! É difícil você ter que deixar seu filho. Um dia já dói, um mês, meu Deus é uma eternidade, passa mais dois meses já é uma loucura, três meses você está fora de si. Você fica fora de si. Ele é uma criança tão forte, forte, bem forte, mas é uma criança que eu olho que está cansado sabe, que eu vejo que* [...] *não sei nem explicar* [...] *ele* [C3] *já ficou várias e várias vezes na beira da morte.* (Mãe 3)

*Quando ele* [C7] *passa mal e não sabemos o que está acontecendo, dá medo ainda, não é 100% segura, no começo foi muito complicado, deu medo, insegurança.* (Mãe 7)

*Ela* [médica] *falou que minha filha ia morrer com um ano e três meses, eu fiquei desesperada porque ela estava com quase nove meses. Com um ano e três meses ela internou bem grave aqui no hospital, todo mundo ficou em estado de choque pensando que ela fosse morrer. Ela ficou um mês e 25 dias na UTI* [*Unidade de Terapia Intensiva*]*, sete meses internada.* (Mãe 12)

O medo da perda do filho era constante e afetava a vida cotidiana dos pais:

*Agora com o ventilador eu consigo dormir um pouco, porque daí ele avisa quando ela* [A4] *não está bem. Antes eu achava mais difícil, porque eu tinha medo de dormir e acordar e ela* [A4] *não acordar mais.* (Mãe 4)

Os relatos expressaram temores relacionados às vulnerabilidades individuais e de caráter biológico e esses sentimentos potencializaram preocupações e sentimentos de apreensão com a saúde de outros membros da família, gerando nos pais mais sofrimento:

*Também penso no meu outro filho, porque se é genético, eu, meu marido, alguém têm.* (Mãe 1)

*Quando eu estava grávida de 6 meses o meu outro filho faleceu com um ano e oito meses, ele também tinha uma síndrome que não foi diagnosticada, por isso que eu corro atrás, estou cobrando todo mundo para ver se tem alguma coisa, porque o problema dele eu acho que pode ser o mesmo problema que o outro teve.* (Mãe 2)

## DISCUSSÃO

O presente estudo mostra que a condição de saúde de uma criança ou adolescente que requer atenção especial desperta sentimentos e experiências vulneráveis para os pais, os quais caminham por um trajeto de medo e solidão, que pode impactar a sua saúde mental.

As vulnerabilidades das famílias de pacientes que necessitam de cuidados complexos e diferenciados por um longo período têm como marco a alta hospitalar, considerando as diversas mudanças implementadas no cotidiano familiar. Estudos identificam que, no momento da alta, há uma transferência da responsabilidade dos cuidados para a família que não possui conhecimentos necessários para o exercício desses cuidados no ambiente domiciliar^(2,7,16)^.

Cuidar do filho, nessa dimensão, exige uma reorganização da rotina doméstica, e isso altera a dinâmica familiar, visto que haverá necessidade de um familiar cuidador em tempo integral, para atender às inúmeras demandas advindas da condição crônica, configurando-se como uma tarefa exaustiva e geradora de sobrecarga. O acúmulo dessas demandas de cuidado ao longo do tempo pode levar muitos pais a experimentar maus resultados de saúde mental, com níveis mais altos de estresse, ansiedade e depressão^(8-9,17)^.

Importante destacar que essa sobrecarga não se relaciona somente às demandas de cuidado, mas aos sentimentos de preocupação com a condição de doença da criança, a impotência e o desconhecimento sobre como cuidar do filho^(7,17-18)^. Embora a condição crônica afeta toda a família, em geral, a mãe por ser a mais envolvida no cuidado, passa a viver para o(a) filho(a), renunciando à própria vida^(19)^. A figura materna, como principal cuidadora das crianças com necessidades de atenção especial à saúde foi evidenciada em inúmeros estudos^(18-23)^, bem como a necessidade de abandono do emprego pelo cuidador familiar principal^(22-23)^, intensificando a vulnerabilidade social, tanto pelo desfavorecimento financeiro, quanto pela falta de socialização com outros indivíduos.

Nesse contexto, compreende-se que diante do diagnóstico do filho, a família se depara com relações vulnerabilizadoras e apresenta sinais de sofrimento em muitas dimensões, seja física, psíquica e social, as quais podem repercutir no cuidado da criança^(2)^. A condição de vulnerabilidade e a necessidade de equilíbrio emocional impactam na saúde mental dos pais, os quais, às vezes, procuram por acompanhamento psicossocial e medicamentoso^(24)^.

Para atender às demandas de atenção à saúde do filho, alguns pais necessitaram abandonar sua vida social, na tentativa de proporcionar o cuidado necessário, mas também para proteger o filho do embaraço social e da humilhação, em decorrência do estigma e preconceito muito presentes ainda na sociedade^(25)^. O preconceito foi destacado por parte dos próprios familiares nessa investigação, e essa atitude levou-os ao afastamento e enfrentamento da situação de forma solitária. Essas situações podem deixá-los mais vulneráveis a desenvolver sofrimento de ordem psíquica e transtorno mental^(26)^.

Um estudo confirmou esses achados ao encontrar que 82,7% das mães apresentavam depressão e, destas, 26,6% depressão grave. Observou-se que quanto maior a limitação funcional da criança e menor a idade, maior foi o nível de depressão entre os cuidadores e que depressão teve um impacto negativo na qualidade de vida dos cuidadores^(23)^. Ainda, pesquisas recentes identificaram que os cuidadores familiares podem elevar o nível de estresse, ansiedade e sintomas depressivos^(10,27)^, sendo que os agravos podem requerer tratamento psicofarmacológico e acompanhamento psicológico^(24)^. Além disso, a situação pode ser considerada como sinalizadora de provável exposição da criança a situações adversas, de insuficiências nas ações de cuidado, devido à limitação dos cuidadores por seu adoecimento^(23,28)^. Outros estudos destacaram que os familiares, inclusive, podem ser afetados fisicamente (privação do sono, cansaço), mental e financeiramente, com impacto negativo a qualidade do atendimento contínuo ao funcionamento da família como um todo^(13,17,25)^.

Nessa perspectiva, é relevante que os profissionais de saúde tenham um olhar diferenciado para os familiares que também adoeceram e precisam de cuidados^(7,25)^, com prioridade ao acolhimento, à escuta qualificada, ao suporte/apoio e às ações de promoção e proteção da saúde^(17,29)^. É preciso evitar criar novas vulnerabilidades, em especial para a cuidadora principal, representada pela mãe neste estudo. Ações e práticas pouco sensíveis às singularidades dessas mães e seus contextos intersubjetivos, podem reproduzir estigmas e discriminações de gênero^(12)^.

Como observado nos resultados, há constante necessidade do apoio para as mães que cuidam de uma criança ou adolescente que necessita de atenção especial à saúde, apoio não apenas do cônjuge, mas também de outros membros da família, amigos e de profissionais de saúde.

O sentimento de culpa pela condição de doença do filho, observado nesta pesquisa, esteve negativamente associado à felicidade e serviu de mediador entre a ansiedade de apego, o apoio e a felicidade. Este sentimento também foi encontrado em estudo com mães de crianças com Síndrome Congênita pelo vírus Zika^(30)^ e paralisia cerebral^(23)^. A família, em sua maioria representada pela mãe, em busca de respostas, atribui para si a responsabilidade pela condição clínica do filho. Um sentimento de culpa e pensamentos de autoacusação podem levar à baixa autoestima^(23)^.

Outro estudo também apontou que a chegada de um filho com deficiência frustra as expectativas e gera sentimentos de fracasso, culpa e tristeza relacionados ao luto pela perda do filho perfeito idealizado^(25)^. Ter sofrido perdas, como a morte de outro membro da família, não importando quanto tempo se passou, faz com que a família se recorde e sinta-se amedrontada pela possibilidade de acontecer novamente. Ainda, ansiedade e o medo de perder o filho surgem como sentimentos que angustiam os pais de crianças com deficiência múltipla e que são causados pela percepção da vulnerabilidade e necessidade de proteger seus filhos^(25)^.

As exigências geradas pelo contexto potencializam, nos pais, a ansiedade, sintomas depressivos, reações de estresse e tensão, sentimento de impotência e de desamparo, que podem culminar no isolamento social e agravar a situação familiar^(10,24)^. Sendo assim, é importante que os profissionais de saúde estejam preparados para orientar e apoiar os mesmos na realização dos cuidados diários, de modo que a ansiedade e o medo sejam minimizados, promovendo a segurança para um cuidado de qualidade para si e para o filho e que, além disso, pode contribuir para redução de readmissões^(31)^.

O uso de estratégias de enfrentamento adaptativas específicas (ou seja, apoio emocional, reenquadramento positivo e aceitação) podem ser promissoras para amparar os pais em situação de vulnerabilidade. Intervenções de apoio por meio da psicoeducação e implementação de comportamentos de enfrentamento podem minimizar o impacto que o isolamento social e as avaliações cognitivas negativas possam ter sobre os resultados de saúde mental de pais e filhos, ampliando os caminhos para cuidar dessas famílias^(10)^.

O modo como as pessoas reagem ao diagnóstico da doença crônica é determinado por suas condições de vida, significados atribuídos à doença, acesso aos recursos disponíveis para o controle do processo saúde-doença, bem como da rede social e apoio recebido. Sendo assim, o reconhecimento e a identificação de vulnerabilidade, por parte da equipe interprofissional, presente na vida da criança e do adolescente que necessitam de atenção especial à saúde, bem como de sua família, é importante, pois pode contribuir no reconhecimento das demandas de cuidado de ambos com vistas ao cuidado integral em saúde^(7,32)^.

Por considerar os efeitos prejudiciais que a saúde mental dos pais pode ter sobre a qualidade do cuidado prestado ao filho, são necessárias intervenções oportunas e efetivas, com vistas a equilibrar as responsabilidades e demandas que acompanham o cuidado de uma criança ou adolescente com necessidades de atenção especial à saúde.

### Limitações do estudo

O fato do estudo ser conduzido no ambiente hospitalar pode ter limitado o conhecimento sobre as necessidades para o cuidado no domicílio. Embora as famílias tenham relatado, é preciso considerar que, ao adentrar o ambiente domiciliar, novas vulnerabilidades para cuidar podem surgir, implicando na necessidade de apoio profissional e comunitário para exercer os cuidados com segurança e autonomia. Nesse sentido, torna-se relevante expandir para outras investigações, tendo como base a observação do cuidado e intervenções para reduzir as vulnerabilidades dessas famílias no cuidado domiciliar.

### Contribuições para área da enfermagem, saúde e de políticas públicas

O estudo contribui para o desenho de estratégias para melhorar o cuidado das famílias de crianças e adolescentes que necessitam de atenção especial, por considerar e identificar sinais que podem intensificar ou criar vulnerabilidades, em especial na dimensão emocional/individual e social, condição que pode fragilizar o cuidado das crianças e dos adolescentes no domicílio, que precisam de cuidado, afeto e dedicação de seus pais para crescerem e se desenvolverem de maneira saudável. Para isso, é preciso uma transformação nos processos de trabalho em saúde, com ações sensíveis às singularidades das famílias, sobretudo das mães, que precisam muito de cuidados por estarem vulnerabilizadas por todo esse contexto.

## CONSIDERAÇÕES FINAIS

A presente investigação identificou que os pais de crianças e adolescentes que necessitam de atenção especial experienciam momentos de vulnerabilidades individuais e sociais para o exercício do cuidado no domicílio, em decorrência da sobrecarga, isolamento e tristeza, principalmente por não receberem o apoio e suporte por parte dos demais familiares. Por vezes, esses mesmos familiares os culpabilizam pela condição de doença do filho, levando-os a acreditar que de fato são os responsáveis pelo filho apresentar necessidades de atenção especial à saúde. Ainda, o medo pela morte do filho ronda suas vidas. Tais vivências podem contribuir para a ocorrência de manifestações que comprometem a saúde mental, expressas por sentimentos de desproteção, ansiedade e depressão.

Por considerar a responsabilidade dos pais e a constante necessidade de atenção dos filhos, é relevante que os profissionais de saúde busquem ampliar as ações de promoção tendo a família como unidade de cuidado, com vistas a reconhecer e reduzir as situações que geram novas vulnerabilidades, ou seja, ameaças, incertezas, preocupações e danos, que podem impactar e fragilizar o cuidado às crianças e adolescentes com necessidades de atenção especial à saúde. Em síntese, é fundamental oferecer aos pais cuidadores o suporte multidisciplinar, em especial na dimensão emocional e social para a promoção da saúde mental dos mesmos.
